# Antenatal Weight Management: Women's Experiences, Behaviours, and Expectations of Weighing in Early Pregnancy

**DOI:** 10.1155/2016/8454759

**Published:** 2016-10-24

**Authors:** J. A. Swift, J. Pearce, P. H. Jethwa, M. A. Taylor, A. Avery, S. Ellis, S. C. Langley-Evans, S. McMullen

**Affiliations:** ^1^School of Biosciences, University of Nottingham, Sutton Bonington, Loughborough LE12 5RD, UK; ^2^National Childbirth Trust, 30 Euston Square, London NW1 2FB, UK

## Abstract

The current emphasis on obstetric risk management helps to frame gestational weight gain as problematic and encourages intervention by healthcare professionals. However pregnant women have reported confusion, distrust, and negative effects associated with antenatal weight management interactions. The MAGIC study (MAnaging weiGht In pregnanCy) sought to examine women's self-reported experiences of usual-care antenatal weight management in early pregnancy and consider these alongside weight monitoring behaviours and future expectations. 193 women (18 yrs+) were recruited from routine antenatal clinics at the Nottingham University Hospital NHS Trust. Self-reported gestation was 10–27 weeks, with 41.5% (*n* = 80) between 12 and 14 and 43.0% (*n* = 83) between 20 and 22 weeks. At recruitment 50.3% of participants (*n* = 97) could be classified as overweight or obese. 69.4% of highest weight women (≥30 kg/m^2^) did not report receiving advice about weight, although they were significantly more likely compared to women with BMI < 30 kg/m^2^. The majority of women (regardless of BMI) did not express any barriers to being weighed and 40.8% reported weighing themselves at home. Women across the BMI categories expressed a desire for more engagement from healthcare professionals on the issue of bodyweight. Women are clearly not being served appropriately in the current situation which simultaneously problematizes and fails to offer constructive dialogue.

## 1. Introduction

The antenatal period is often considered to be an important opportunity for health promotion and guidance, offering the so-called “teachable moments” [1]. Usual antenatal care associated with normal pregnancy puts women into greater contact with health professionals [2], and it is thought that responsibility for their developing baby can be an important motivator for behaviour change [1]. Pregnancy-related physical changes can also be said to cause a renegotiation of women's identity towards functionality and mothering [3], explaining why women can become more perceptive to health education [4].

In the context of maternal obesity, the UK's National Institute of Health and Care Excellence (NICE) recommends that the main focus of weight loss lies in the periconceptual period and that during pregnancy a woman should receive advice on healthy lifestyles and weight* management* [5]. Weight gain during pregnancy and changes in shape can be considered both expected and healthy [6], and NICE does not recommend weight loss during pregnancy as it may pose a risk by impairing foetal nutrition [5]. However, the UK does not have any formal, evidence-based recommendations for amount of gestational weight gain, although a guidance range of 10–12.5 kg (22–26 lbs) is used by NHS England [7] and has been recommended by the Department of Health in the past [8]. In contrast the USA has specific recommendations for weight gain for different BMI groupings published in the Institute of Medicine's “Weight Gain During Pregnancy: Reexamining the Guidelines” [9]. Women with a BMI of 20–25 kg/m^2^ are advised to gain 11–16 kg during pregnancy, whereas women with obesity are recommended to limit weight gain to no more than 9 kg. Since the late 1990s regular weighing has not been encouraged in the UK [10] and the current NICE [5] advises weighing women only at booking (usually ~10 weeks of gestation), while a joint guideline from the Centre for Maternal and Child Enquires and the Royal College of Obstetrics and Gynaecology recommended follow-up weighing in the 3rd trimester only if the women has a BMI that can be classified as obese at booking [11]. Despite recent evidence that pregnant women with BMI 18–29.9 kg/m^2^ found regular weighing to be acceptable and useful [12] and the observation that monitoring can be reassuring [13], there remain fears that this practice would draw (negative) attention to the body, fuelling body image problems [14], particularly among those with internalised weight stigma.

There is also a lack of detailed, evidence-based guidance for clinicians on* how* to achieve appropriate weight gain. NICE [5] recommends that practitioners adopt a patient-centred approach, asking women if they would like advice about their weight and if so when they would like to receive it. This assumes that weight and weight gain have already been defined by the practitioner and women as a topic in need of discussion, but no guidance is given as to how—or indeed whether—the practitioner should take responsibility for raising the issue.

This lack of guidance is positioned within a society that has a significant pro-thin bias [15] and substantial anti-fat attitudes [16]. Women may be increasingly concerned about weight gain during pregnancy [17] and aware of the health risks associated with higher weights [18], but this can be opposed by the general acceptance of the inevitability of gestational weight gain (as opposed to weight gain outside of pregnancy) which temporarily exempts women from adherence to ideal [3]. Taken together it is, therefore, not surprising that there is confusion, contradiction, distrust, and negative effects associated with antenatal weight management interactions—both on the part of practitioners and on pregnant women [19]—along with ambivalence among midwives who are considering both women-centeredness and risk management as priorities [20].

The current study—the MAnaging weiGht In pregnanCy (MAGIC) study—sought to examine women's experiences of routine antenatal weight management provision in Nottingham, where the prevalence of obesity in pregnant women is 20% higher compared to England as a whole [21, personal communication]. In addition to giving a perspective on local needs, this observational study extends previous research by recruiting a cohort of women in early pregnancy and collecting follow-up data (until 12 months postpartum) on a wide range of biological, psychological, social, and behavioural factors. This allows an examination of an objective weight measurement (at baseline) as a dynamic variable subject to individual, ongoing appraisal. It also allows consideration of how advice on weight is positioned alongside advice on diet and physical activity, as well as assessments of dietary and physical activity behaviour, through the whole antenatal and postnatal period. The current analysis uses quantitative and qualitative data collected at recruitment (baseline) only and aims to describe the sample's experiences, behaviours, and expectations of antenatal weight management in early pregnancy.

## 2. Materials and Methods

### 2.1. Ethical Approval

This study was approved by the NHS Health Research Authority (NRES Committee East Midlands) and Nottingham University Hospitals NHS Trust, Research and Innovation Department (12/EM/0267).

### 2.2. Sample and Recruitment

Women were recruited from the antenatal clinic at Queens Medical Centre (QMC, Nottingham University Hospitals NHS Trust) while waiting for either their “dating scan” (an ultrasound scan, usually between 10 weeks 0 days and 13 weeks 6 days, to determine gestational age) or their “18–20-week anomaly scan” (ultrasound screening for structural anomalies, normally between 18 weeks 0 days and 20 weeks 6 days), both of which are routine appointments for all women according to NICE antenatal care pathway [2]. Women aged 18 years or over and of any sociodemographic background, bodyweight, and parity were approached by a researcher and provided with information about the study. Once they had read the information and if they agreed to take part, written consent was obtained. No incentive was offered.

### 2.3. Measures

Participants completed a paper-based questionnaire collecting data on a number of social, physiological, psychological, and behavioural measures. The variables used in the current analysis were as follows. (1) Sociodemographics: participants self-reported their age, ethnicity, gestation and number of embryos of current pregnancy, and number of other children. In addition, participants self-reported their own and partner's (if applicable) occupation, which were coded using the Standard Occupation Classification 2010 and then classified using the National Statistics Socioeconomic Classification (rebased on SOC2010; NS-SEC) [22]. To assess the socioeconomic status of the household, the highest reported NS-SEC score was taken as the Household Reference Point. (2) Anthropometrics: measurements of weight and height were taken by trained researchers on calibrated equipment (Leicester Height Measure, Marsden, UK, and bathroom scales, Salter, UK). Body Mass Index (BMI) was calculated using the standard formula (kg/m^2^) and classified using the World Health Organization's criteria (underweight <18 kg/m^2^, recommended weight 18–24.9 kg/m^2^, overweight 25–29.9 kg/m^2^, and obese ≥30 kg/m^2^) [23]. Participants were also asked to self-report their prepregnancy weight in stones and pounds or in kilogrammes and describe how this prepregnancy weight was measured with the options “bathroom scales,” “measured on scales by a midwife, by GP, and at a hospital appointment,” “I have guessed my weight,” and “other.” (3) Weight monitoring behaviour and advice: participants were asked to report whether they had been weighed and by which healthcare professional during their current pregnancy and whether they had received specific advice about their weight following being weighed. Open questions were asked to women to describe the advice received following being weighed and how they felt about being weighed and any subsequent advice. Participants also responded to the question “which statement best describes what you were doing at the moment?” with the options “trying to lose weight,” “trying to keep my weight at the same level,” “not trying to do anything about my weight,” and “trying to put on weight.” (4) Current shape concern and antenatal weight change expectations: shape concern was assessed using 7 items from the shape concern subscale of the Eating Disorders Examination Questionnaire Version EDE-Q [24]. The item “have you felt fat?” was omitted due to its multidimensionality and value-laden terminology. A summative score was calculated using the mean of all 7 items; scores ranged 0–6 with higher scores indicating more shape concern. Cronbach's alpha for the 7 items in the current sample was 0.91, indicating internal consistency [25]. Participants were also asked whether they expected their weight to change and if so in what direction and by how much. (5) Awareness of guidance and sources of information: participants were asked whether they were aware of the Department of Health's guidance around weight gain and if so what this was. Participants reported what they perceived to be the main sources of information around bodyweight, diet, and exercise, and an open question was asked to women to describe what they thought about sources of information available.

### 2.4. Data Analysis

Quantitative data were analysed using SPSS version 22 (SPSS Inc., Chicago, IL, USA). Data entry was conducted by three members of the research team and all data entry was double-checked by another member of the team. The dataset was inspected for univariate outliers and missing data. Normality of continuous variables was assessed using the Kolmogorov–Smirnov test, and appropriate parametric and nonparametric statistics were then used to describe the sample. Chi-squared and Kruskal-Wallis tests were used to investigate the relationship between weight classification at recruitment and receiving advice and shape concerns, respectively. These were followed by* post hoc *2 × 2 chi-squared tests and Mann-Whitney *U* tests as appropriate. The relationship between shape concerns and amount of weight women expected to gain during pregnancy was analysed using Spearman's rank correlation. Qualitative data from open questions were subjected to an inductive, descriptive content analysis [26].

## 3. Results

### 3.1. Sociodemographics

The research team approached 786 women in clinic and 360 consented to participate, were weighed and measured, and took the study materials home with them. Questionnaires were returned by 193 women and these women were considered to be recruited onto the study. At recruitment the participants' age was normally distributed with a mean of 32.8 years (SD 5.2 yrs, min 18.9 yrs, and max 47.1 yrs). 86% (*n* = 166) of the sample self-identified with a white ethnicity, 94.6% (*n* = 181) were living in a household with at least the equivalent of one full-time salary, and 79.6% (*n* = 121) were living in a household with a Household Reference Point of 1-2 (data were missing on ethnicity and occupation for 4 and 2 participants, resp.).

Participants' self-reported gestation was between 10 and 27 weeks with 41.5% (*n* = 80) participants in weeks 12–14 and 43.0% (*n* = 83) in weeks 20–22. The majority were expecting a singleton (*n* = 177, 91.7%). 43.5% (*n* = 84) of the sample were primiparous, 40.9% (*n* = 79) had one child, and 15.5% (*n* = 30) had two or three children.

### 3.2. Anthropometrics

At recruitment participants' Body Mass Index (BMI) had a non-Gaussian distribution with a median of 25.1 kg/m^2^ (IQR 6.5 kg/m^2^, min 17.5 kg/m^2^, and max 53.5 kg/m^2^), and 50.3% (*n* = 97) of the sample could be classified as overweight or obese (Table 1). There were no significant differences in terms of recruitment BMI between participants and those 167 women consented but did not return the study materials (median 25.6 kg/m^2^; IQR 7.4 kg/m^2^, min 16.4 kg/m^2^, and max 47.6 kg/m^2^). Self-reported prepregnancy weights were available for 168 women and had a median of 22.8 kg/m^2^ (IQR 5.5 kg/m^2^, min 15.9 kg/m^2^, and max 51.3 kg/m^2^). Women were most likely to take measurements using bathroom scales (66.7%, *n* = 112) while 23.2% (*n* = 39) were based on measurements taken by a healthcare professional. Women had, on average, gained 0.26 kg/wk (IQR 0.34 kg/wk, min −1.05 kg/wk, and max 9.83 kg/wk) since conception.

Among the 168 women who had complete data on both variables, 54 self-reported a prepregnancy weight that could be classified as overweight or obese using measurements taken at recruitment (Table 1). However, 26 of the 114 women who self-reported a prepregnancy weight that could be classified as underweight or recommended weight were classified as overweight or obese using measurements taken at recruitment. Further analysis revealed that 20 of these 26 women were recruited at gestation 20–22 weeks (76.9%) and the remainder were recruited between 12 and 19 weeks.

### 3.3. Weight Monitoring Behaviour and Advice

95.3% (*n* = 184) of women reported having been weighed by a healthcare professional during their current pregnancy, most commonly a midwife (*n* = 181). 29 of these 184 women (15.8%) reported that they had received specific advice about their weight (Table 2). There was a significant association between receiving advice and weight classification at recruitment (*χ* ^2^_(2)_ = 9.57, *p* < 0.001; the one woman with BMI < 18 kg/m^2^ was removed from this analysis due to insufficient cell count). Women who could be classified as obese were significantly more likely to receive specific advice about their weight after being weighed, compared to women who could be classified as having a recommended weight (*χ* ^2^_(1)_ = 9.04, *p* < 0.01) or overweight (*χ* ^2^_(1)_ = 4.20, *p* < 0.05) at recruitment. Content analysis of the advice reported by participants who had received comments about weight from health professionals covered a range of themes, as did women's feelings about being weighed (Table 2).

40.4% (*n* = 78) of participants reported that they had weighed themselves during their current pregnancy, and the majority of these weighed themselves weekly or fortnightly (57.7%, *n* = 45). The majority of women reported that they were not trying to do anything about their weight at the moment (*n* = 142, 73.6%) while 19.2% (*n* = 37) were trying to keep the same weight. Women with a BMI at recruitment that could be classified as overweight or obese were significantly more likely to be trying to keep the same weight, compared to women with a BMI < 25 kg/m^2^ (*χ* ^2^_(1)_ = 6.65, *p* < 0.05). The two women who reported trying to lose weight both had a BMI at recruitment that could be classified as obese.

### 3.4. Current Shape Concern and Antenatal Weight Change Expectations

There was a significant association between BMI classification at recruitment and shape concerns (*χ* ^2^_(2)_ = 19.71, *p* < 0.001). Women with a BMI at recruitment which could be classified as a recommended weight had significantly lower shape concern scores than women with a BMI which could be classified as overweight (*Z* = −3.35, *p* < 0.01) or obese (*Z* = −3.85, *p* < 0.001). There were no significant differences between women with a BMI which could be classified as overweight and obese (Table 3).

None of the women reported that they expected to lose weight during pregnancy while 1.6% (*n* = 3) expected no change in their weight, and 5.7% (*n* = 11) reported that they had no idea what to expect. 50.3% (*n* = 97) reported that they were expecting to gain weight but were not able quantify it, while 42.5% (*n* = 82) of the sample were able to quantify their expected weight gain (median 10.1 kg, SD 4.58 kg, min 2.27 kg, and max 22.23 kg; excluding one woman with multiple pregnancy who provided that data on this variable did not significantly alter the distribution). There was no significant association between those women who expected no weight change, weight gain (but not quantified), and quantified weight gain and BMI classification at recruitment. There was small, significant, positive correlation between shape concerns and amount of weight women expected to gain during pregnancy (*r*_*s*_ = 0.34, *p* < 0.01, and *n* = 82).

### 3.5. Awareness of Guidance and Sources of Information

39.4% (*n* = 76) of the sample reported that they were aware of guidance around weight change during pregnancy, and 59 women reported that guidance recommended a weight gain of 11.3 kg (IQR 4.2 kg, min 2 kg, and max 19.1 kg). 80.8% of the sample reported that healthcare professionals were main sources of information, 79.3% print and online information, and 37.8% family and friends. Women's responses to the open question about what they thought about sources of information available (*n* = 137) were often lengthy (max 157 words). Women across the BMI categories were more likely to report that sources of information were adequate or good rather than insufficient, and the thematic content analysis revealed three themes: general adequacy, healthcare professionals' role, and lay sources (Table 4).

## 4. Discussion

This study reports the broad range of women's experiences, behaviours, and expectations of routine antenatal weight management provision in Nottingham. In line with the recommendations of the NICE [5] guidelines and similar to a small UK study by Brown and Avery [27], most women in the sample reported having been weighed in early pregnancy and by a midwife. It is, however, notable that a low proportion of women weighed reported having received advice after these measurements—even less than that observed by McDonald et al. [28]. Presumably practitioners are using the information to refer higher weight women to consultant-led care, and from an ethical perspective women should be aware of why these measurements are being taken and how it will be used to plan their care. However, NICE also recommended that women with a BMI over 30 should be referred to a dietitian or appropriately trained professional to receive personalised advice on healthy eating and physical activity. Although significantly more women of a higher weight received advice, excessive gestational weight gain can occur regardless of prepregnancy BMI classification. It has been suggested that midwives are optimally placed to deliver advice on gestational weight gain [29] and that as they deal with sensitive issues and women's anxieties as a core part of their role, they are very well equipped [12]. Indeed, NICE recommended discussion of how to achieve a healthy lifestyle during antenatal contacts for all pregnant women. However, other researches reveal that midwives fear offending and alienating women by discussing the issue early in the therapeutic relationship [30]. These are valid concerns due to the moralistic nature of weight, reports of stigma in antenatal settings [18, 30–32], and in the current sample women both of higher and lower weights were embarrassed. However, women in this sample who were weighed and received advice were generally not negative about the experience and more similar to the views expressed by women in research by Olander et al. [33]. The language employed by women in this sample could not be said to be overwhelmingly positive either—rather the tone was one of confirmation of something uncontroversial.

Taken together the majority of women in the sample (regardless of BMI) did not express any barriers to being weighed and as 40.8% reported weighing themselves at home on scales, there is some justification for providing women with an opportunity to take accurate measurements using reliable equipment under supervision. This phenomenon of self-monitoring has been reported elsewhere; for example, women who disengaged from an antenatal weight service cited confusion and disappointment about not being weighed regularly [34, 35] and reported self-monitoring in half of the sample of women which included those who exhibited both recommended and excess gestational weight gain. What is less clear are women's motivations for regular weighing. It is perhaps being used as a means to motivate behaviour as suggested by Daley et al. [12] but—due to the lack of agreed targets—it might also reflect women's scientific curiosity and fascination as to their new-found functionality [3]. This would explain the relatively low levels of shape concern seen in the sample, even among higher weight women. Tiggemann [36] describes that while body image might be a relatively stable construct, the importance vested in it is dynamic.

Considering the lack of dialogue between women in this sample and their practitioners, it is perhaps unsurprising that the majority are unable to recall the guideline expectations for weight gain used by NHS England and the Department of Health or what to expect during their own pregnancy beyond a sense that they will “gain weight.” Those women who did have an expected weight gain that could be quantified varied widely but were, on average, consistent with 10–12.5 kg.

When asked about the advice generally available on weight, diet, and exercise, participants used more positive than negative comments. However, a deeper examination revealed several narratives. In line with previous work [18, 27, 32, 33, 35, 37], women did not feel that their weight or indeed diet and exercise were priorities for midwives and other healthcare professionals. In the current study practitioners detached from the subject by employing terminology such as “BMI” and actions such as “keep an eye on [your weight].” Women also reported that there were not ready opportunities to ask questions about “nonroutine” or “nonemergency” topics. This perhaps also accounts for the equal reliance on Internet sources as “main sources of information,” despite the awareness of their limitations. Olander et al. [33], Arden et al. [31], and Brown and Avery [27] have also described how gaps in knowledge on weight can be filled using self-study.

For those who had accessed advice, there was frustration that it was too general, not personalised, and diet-focused. Women are not, therefore, perceiving the advice to be “practical and tailored” as recommended by NICE [5]. Similarly, Heslehurst et al. [38] described how dietary information was provided* ad hoc* and not linked to weight management, while Brown and Avery [27] report that many participants stated advice was brief and lacking in detail. Interestingly women of a higher weight reported that the advice they received was too idealistic and not supported by advice on process.

In contrast to those who want to be better informed, there are women who actively avoided information about weight, diet, and exercise. The issue of bodyweight was sometimes deemed to be not salient (at all or due to time at which it is received) and previously authors have reported women preferring to wait until after birth [34, 39]. Worryingly advice from practitioners was in some cases dismissed as unreliable and Arden et al. [31] also describe how women can lack trust in “official” advice. Others reported a wish to avoid potential negative emotions which once again speaks to the value-laden nature of bodyweight.

### 4.1. Strengths and Limitations

As with previous work in the UK (e.g., [31]), the current sample is not wholly representative of the population. It had twice the proportion of women from a household with an NS-SEC score of 1 or 2 compared to the census data for the East Midlands (<65 yrs) [40], and the average age of mothers (32.8 yrs) was also higher than the 30.0 years reported in the Office for National Statistics data [41]. However, the majority of women were recruited at 12–14 weeks of gestation and 20–22 weeks of gestation which reflects the function of the clinics recruited from (namely, the 10–12-week dating scan and 18–20-week anomaly scan), and higher weight women were represented at a level similar to that from national statistics (i.e., 50% overweight or obese at the start of pregnancy [41]). It is interesting to observe that higher weight women were not systematically deterred from participation due to the objective weight and height measurements taken by the researchers, but when taking into account participants' low body shape concerns it may be that women (across the BMI categories) with body image and weight concerns may be underrepresented. This limits the generalisability of the findings and it would be inappropriate to conclude that weighing is generally acceptable across the socioeconomic spectrum and in various ethnic identities. However, the findings do reveal an unmet need for engagement on the issue of bodyweight among some women across the BMI categories.

The uses of BMI categories to identify obesity, indicate risk, and decide upon care are controversial but are widely used in research and clinical practice. Measurements can be taken throughout pregnancy, from prepregnancy [42, 43] to the 3rd trimester [44]. The mismatch between BMI figures in Table 1, calculated using both the self-reported prepregnancy and measured recruitment weights, is possibly due to misreporting and/or gestational weight gain, and it is not possible with the current study design to separate out these potential influences.

## 5. Conclusion

The positioning of prepregnancy bodyweight and gestational weight gain as* problematic* in the national consciousness has for many years been encapsulated in guidelines such Department of Health, National Institute of Health and Care Excellence, and the Royal College of Obstetrics and Gynaecology. Indeed, the focus has intensified of late, most recently with comments from the Chief Medical Officer [45] who described obesity as the “biggest threat to women's health” and the subsequent media coverage. It is, therefore, unsurprising that the current study revealed a desire for engagement on the issue of bodyweight among some women across the BMI categories. However, the lack of specific guidelines, the lack of available support around process, and the reluctance of some practitioners to engage in this complex and value-laden topic are all barriers.

Women are clearly not being served appropriately in the current situation which simultaneously problematizes and fails to offer solutions. Given the weight of medical opinion that bodyweight should be an issue to be addressed during pregnancy, future work needs to move away from the current obstetric risk management framework to an empowerment approach [6] and build the capacity of practitioners to deliver individualised weight-related advice without prejudice. Arguably the antenatal period offers a unique opportunity to counter the current negative reductionist dialogue around weight gain with one that emphasises the body's capabilities. Specific behavioural guidelines and positive framed advice could be developed and applied in a flexible, nonjudgmental manner to offer reassurance and empowerment.

## Figures and Tables

**Table 1:** Body Mass Index (BMI) classifications of participants calculated using weight measured at recruitment and self-reported prepregnancy weight.

	BMI^*∗*^ calculated using weight measured at recruitment (*n* = 193)	BMI^*∗*^ calculated using self-reported prepregnancy weight (*n* = 168)
BMI < 18 kg/m^2^	2 (1%)	7 (4.2%)
BMI 18–24.9 kg/m^2^	94 (48.7%)	107 (63.7%)
BMI 25–29.9 kg/m^2^	61 (31.6%)	33 (19.6%)
BMI ≥ 30 kg/m^2^	36 (18.7%)	21 (12.5%)

^*∗*^Both BMI calculations used height assessed at recruitment.

**Table 2:** Weight advice received by participants after being weighed, by BMI classification at recruitment.

	Weight advice received (*n* = 29)	Themes of feelings about being weighed and advice received^*∗*^ (*n* = 26)	Themes of weight advice received after being weighed^*∗*^ (*n* = 27)
BMI < 18 kg/m^2^	1 (3.4%)	Embarrassed (*n* = 1)	Advised that BMI required consultant-led care (*n* = 1)

BMI 18–24.9 kg/m^2^	13 (44.8%)	Grateful/happy (*n* = 3) Fine/did not mind (*n* = 7) Embarrassed (*n* = 1)	Advised that BMI is “low” (*n* = 3) Advised that BMI is “healthy” (*n* = 3) Recommended healthy diet (*n* = 4) Emphasised need for weight gain (*n* = 2) Emphasised need for monitoring (*n* = 1)

BMI 25–29.9 kg/m^2^	4 (13.8%)	Very sensible (*n* = 1) Fine (*n* = 1)	Advised not to lose weight but maintain (*n* = 1) Recommended avoidance of “sugary & fatty” foods (*n* = 1)

BMI ≥ 30 kg/m^2^	11 (37.9%)	Fine/did not mind (*n* = 4) Grateful/happy (*n* = 4) Shocked but reassured (*n* = 1) Sceptical of advice (*n* = 1)	Advised to maintain weight/avoid weight gain (*n* = 3) Recommended healthy diet (*n* = 2) Recommended exercise (*n* = 1) Recommended commercial weight loss organization (*n* = 2)

^*∗*^Themes are not mutually exclusive, and some responses could not be coded as they did not provide a description of the specific advice received.

**Table 3:** Shape concern subscale scores by BMI classification at recruitment.

	Median	Interquartile range	Min	Max
BMI 18–24.9 kg/m^2^	0.86	1.29	0.14	5.71
BMI ≥ 25–29.9 kg/m^2^	1.71	1.86	0.14	5.00
BMI ≥ 30 kg/m^2^	2.14	2.29	0.14	4.71

**Table 4:** Participants' feelings about sources of information during pregnancy on weight, diet, and exercise, by BMI classification at recruitment.

Themes^*∗*^	BMI < 18 kg/m^2^ (*n* = 1)	BMI 18–24.9 kg/m^2^ (*n* = 69)	BMI ≥ 25–29.9 kg/m^2^ (*n* = 43)	BMI ≥ 30 kg/m^2^ (*n* = 24)
*General adequacy*				
Generally fine/good/plenty		14 (20.3%)	11 (25.6%)	4 (16.7%)
Generally not sufficient		8 (11.6%)	4 (9.3%)	2 (8.3%)
Not salient in very early pregnancy	1		3 (7.0%)	
Not salient until postpartum				3 (12.5%)
Emphasis on diet, not weight			5 (11.6%)	
Too general/no guidelines		13 (18.8%)	4 (9.3%)	2 (8.3%)
Individualised advice preferred		6 (8.70%)		
To idealistic			1 (2.3%)	1 (4.2%)
No information on *how* to change			4 (9.3%)	1 (4.2%)
No information on *why* to change			2 (4.7%)	1 (4.2%)
*Healthcare professionals role*				
Do not appear concerned		3 (4.3%)	3 (7.0%)	1 (4.2%)
Information can be confusing/unreliable/conflicting			3 (7.0%)	3 (12.5%)
Have to ask/seek information		7 (10.1%)	6 (14.0%)	3 (12.5%)
More active engagement preferred		3 (4.3%)	1 (2.3%)	
Subject too personal for HCP		2 (2.90%)	2 (4.7%)	
Do not seek/avoid information		9 (13.0%)		4 (16.7%)
*Lay sources*				
Happy with information available via the Internet/apps/magazines/books		5 (7.2%)		
Information can be confusing/unreliable/conflicting		8 (11.6%)	1 (2.3%)	3 (12.5%)
NHS web resources good/reliable	1	8 (11.6%)	2 (4.7%)	
Better signposting required		1 (1.4%)		

^*∗*^Themes are not mutually exclusive.
